# Severe acute respiratory coronavirus virus 2 (SARS-CoV-2) vaccine acceptance in employees in an integrated health system in the Midwest

**DOI:** 10.1017/ash.2021.166

**Published:** 2021-07-23

**Authors:** Hayden L. Smith, Maura J. Prescher, Katherine R. Sittig, Lisa A. Veach

**Affiliations:** 1 UnityPoint Health–Des Moines, Des Moines, Iowa; 2 UnityPoint Health System Services, Des Moines, Iowa

Vaccine hesitancy, defined as a “delay in acceptance or refusal of vaccination despite availability of vaccination services,” remains a significant barrier to vaccine efforts in the United States.^1^ For some subpopulation groups, vaccine hesitancy has been a long-standing obstacle for social and complex historical reasons.^2^ Hesitancy may extend to the severe acute respiratory syndrome coronavirus 2 (SARS-CoV-2) vaccine. It is important to understand variables associated with vaccine acceptance, particularly among healthcare workers (HCWs) because high vaccination rates can mitigate the impact of the virus. In this study, we surveyed employees of an integrated health system in the Midwest United States regarding SARS-CoV-2 vaccine acceptance.

## Methods

### Participants and setting

An electronic-based survey was distributed within a Midwest US health system. The study included 4 hospitals (ie, adult level 1 trauma center, 2 adult level 4 trauma centers, and a pediatric level 2 trauma center) and their associated clinics within a single metropolitan area. The survey link was e-mailed to all employees, regardless of full-time status or role. Employees completing the survey were eligible to receive an antibody test for SARS-CoV-2 at no cost.

### Survey

The survey contained questions about demographics, occupation, SARS-CoV-2 exposures, and prior testing (Supplementary Document 1 online). Participants who completed the survey were sent a short subsequent survey about new SARS-CoV-2 exposures, infections, and vaccine status. The initial survey was available from December 4, 2020, through January 3, 2021, and the subsequent survey was available from December 16, 2020, through January 29, 2021.

### Statistical analysis

Participant characteristics were examined in relation to vaccine acceptance. Vaccine acceptance was defined by pooling the following responses to “Have you received the SARS-CoV-2 vaccine outside of a clinical trial?”: “Yes, 2 doses”; “Yes, 1 dose”; “No, but I intend to” (vaccine acceptance), “No, and I am unsure” (hesitancy), and “No, and I don’t intend to” (refusal). Hesitancy and refusal were collapsed together as the reference group in a generalized linear model fit with a binomial distribution and logit link function. Estimates are reported as adjusted odds ratios (ORs) with 99% confidence intervals (99% CIs).

Vaccine eligibility for direct HCWs within the health system began 2 weeks after the study survey was made available. For subjects with a subsequent survey completed ≥3 weeks after vaccine eligibility, their planned vaccine response was compared to self-reported vaccine status at the time of the subsequent survey. The study was approved by the institutional review board (no. IM2020-126), including a waiver of written consent for survey response data.

## Results

Of the 6,009 employees sent a study invitation, 2,848 (47%) completed the electronic survey and 2,118 (35%) received a SARS-CoV-2 antibody test. Sample characteristics stratified by vaccine acceptance are presented in Table 1. Overall, 51% gave a response classified as vaccine acceptance, 36% as vaccine hesitancy, and 13% as vaccine refusal. Descriptive results revealed age, gender, occupational group, and self-reported low likelihood of COVID-19 as potentially associated with vaccine acceptance.

**Table 1.** Participating Employee Characteristics by SARS-CoV-2 Vaccine Acceptance Status in a Midwest Health System (n = 2,848)

Characteristic	Vaccine Acceptance Status, No. (%)	
	Yes^a^ (n=1,446)	No (n=1,402)
**Age, y^b^**		
21–30	280 (43)	375 (57)
31–40	379 (45)	471 (55)
41–50	331 (53)	296 (47)
51–60	286 (61)	186 (39)
≥61	159 (71)	65 (29)
**Sex^c^**		
Female	1,183 (48)	1,296 (52)
Male	257 (71)	103 (29)
**Work status**		
Full time	1,204 (51)	1,151 (49)
Part time	242 (49)	251 (51)
**Primary work setting**		
Inpatient/Emergency department	805 (51)	762 (49)
Outpatient	464 (51)	453 (49)
Nonclinical	177 (49)	187 (51)
**Occupation group**		
Physicians	183 (86)	29 (14)
PA/NP	64 (57)	48 (43)
Nurses, nursing assistant	596 (46)	697 (54)
Allied health	264 (49)	267 (50)
Support roles	152 (49)	157 (51)
Other nonpatient care	187 (48)	204 (52)
Provided direct patient care (Yes)	1,101 (51)	1,039(49)
**Patient population^d^**		
Adult	562 (53)	503 (47)
Pediatric	190 (57)	146 (43)
Both	349 (47)	390 (53)
Provide Care to Patients Undergoing AGPs^d^	401 (50)	396 (50)
**How likely do you think it is that you’ve had COVID-19?**		
Extremely unlikely	102 (66)	52 (34)
Unlikely	484 (61)	314 (39)
Equally likely and unlikely	547 (48)	597 (52)
Likely	149 (40)	221 (60)
Extremely likely	164 (43)	218 (57)
Previously tested for COVID-19 (Yes)	854 (51)	833 (49)
If yes, was test positive?	142 (46)	170 (54)
**How do you think you got COVID-19?**		
Unsure	28 (33)	58 (67)
Work exposure from patient	39 (51)	37 (51)
Home exposure	35 (51)	33 (49)
Community exposure	26 (54)	22 (46)
Work exposure from coworker	14 (42)	19 (58)
Prefer not to answer	0 (0)	1 (100)

Note. PA/NP, physician assistant/nurse practitioner; AGP, aerosol-generating procedure.

a Includes 38 employees that already received a first dose of the vaccine.

b 20 subjects under 21 years of age not listed.

c 9 employees reported another category for gender.

d Subsample of employees reporting direct patient care.

Based on study modeling, men had a 1.05 (99% CI, 1.01–1.10) times greater adjusted odds of self-reported vaccine acceptance than women. Nurses and nursing assistants had the lowest vaccine acceptance rate. Physicians had a 1.11 (99% CI, 1.03–1.19) times greater adjusted odds of acceptance than the nurse and nursing-assistant group and a 1.10 (99% CI, 1.03–1.18) times greater adjusted odds of vaccine acceptance than all other groups combined. Within the physician category, acceptance rates were balanced across genders with 88% male and 85% female physician acceptance. We detected a positive trend for greater acceptance across the increasing age groups (*P* < .0001). This trend was further demonstrated by employees aged 51–60 years; they had a 1.06 (99% CI, 1.01–1.11) times greater adjusted odds of acceptance than the group aged 21–30 years. Those aged >60 years had a 1.08 (99% CI, 1.01–1.16) times greater adjusted odds of acceptance. Finally, we detected an inverse trend across responses to likelihood of having had coronavirus disease 2019 (COVID-19); those self-reporting a low likelihood had greater vaccine acceptance (*P =* .0017). Post hoc analyses failed to reveal any multiplicative interactions between gender, age, and job category for vaccine acceptance.

Table 2 reports discordance between initial planned vaccine hesitancy and refusal by job category versus self-reported actual vaccination status for HCWs with an available subsequent survey. The results revealed some employees initially reported planned hesitancy or refusal but then self-reported having received at least 1 dose of a SARS-CoV-2 vaccine. This occurred in 61% of physicians, physician assistants, and nurse practitioners; 37% of the allied health group; and 29% of the nurse and nursing-assistant group.

**Table 2.** Self-Reported Planned Vaccine Acceptance/Hesitancy for Direct Healthcare Providers^a^

Planned Vaccine Response	Self-Reported Receiving at Least 1 Vaccine Dose by Job Category, No./Total (%)				
	Physicians	Physician Assistants/Nurse Practitioners	Nurses/Nursing Assistants	Allied Health	Totals
Acceptance	57/70 (81)	16/24 (67)	155/210 (74)	65/90 (72)	293/394 (74)
Hesitancy	7/9 (78)	3/10 (30)	65/197 (33)	27/59 (46)	102/275 (37)
Refusal	3/3 (100)	1/1 (100)	10/63 (16)	7/34 (21)	21/101 (21)
Totals	67/82 (90)	20/35 (57)	230/470 (49)	99/183 (54)	416/770 (54)

a From survey compared to self-reported vaccination status from short post-surveys completed 21–46 days after participant became vaccine eligible (n=770). Excludes subsequent surveys completed ≤21 days after becoming vaccine eligible. The use of 21 days allowed eligible employees time to receive a dose and the 46-day cap represents the last day the subsequent survey was available for completion. Hesitancy and refusal categories were collapsed in the calculation of estimates in the body of the manuscript.

## Discussion

In this study, factors associated with SARS-CoV-2 vaccine acceptance were identified in a sample of nearly 3,000 employees in a health system in the Midwest. More than half of surveyed employees reported planned vaccine acceptance. Male sex, older age, low likelihood of prior COVID-19, and occupation as a physician were independently associated with vaccine acceptance. However, a proportion of employees who had initial vaccine hesitancy or refusal reported receiving at least 1 dose of SARS-CoV-2 vaccine.

The 51% vaccine acceptance rate was consistent with published surveys of American HCWs, which have shown 36%–58% acceptance rates for the vaccine.^3–5^ The presented study supports prior findings that male gender and older age may be associated with vaccine acceptance among HCWs.^3–5^ Fewer studies have compared occupational groups, but available data suggest that physicians have a higher rate of vaccine acceptance.^6,7^

Published study results have been mixed regarding associations between direct patient care and vaccine acceptance. Some show higher acceptance in HCWs providing direct patient care,^6^ and others show an opposite relationship.^3,4^ Our results indicate no association between direct patient care and planned vaccine status. Among direct patient care providers, no differences were detected between inpatient and emergency room providers compared with outpatient providers. Also, vaccine acceptance status was balanced among those providing care to patients undergoing aerosol-generating procedures (AGPs).

A study of Philadelphia HCWs by Kuter et al^3^ showed that a prior COVID-19 test was associated with higher vaccine acceptance. The present study did not reflect this association. In addition, our study revealed greater vaccine acceptance in participants self-reporting a low likelihood of previously having had COVID-19. Speculatively, this relationship could be due to a perceived lack of need for SARS-CoV-2 vaccine if one has already had COVID-19 or because those who are more risk averse may be more likely to accept the vaccine.

The presented study differs from other reports because the HCW population was a large, nonacademic healthcare system with a significant portion of respondents based in the outpatient setting. In addition, the study is the first to survey HCWs from the Midwest United States. Also unique to the study is the examination of how initial vaccine acceptance, hesitancy, or refusal are related to eventual reported uptake of the SARS-CoV-2 vaccine. These data underscore the need to support SARS-COV-2 vaccine efforts targeting both hospital- and clinic-based HCWs as well as those who have previously had COVID-19 by demonstrating that initial vaccine hesitancy or refusal can change in a short period.

The study has several limitations. Study data were based on self-reported survey responses, and not all eligible employees participated. Participants completing the survey and a subsequent survey later in the study could systematically differ from those completing them earlier. Employees who reported not receiving the vaccine on the subsequent survey could have gone on to receive it. Employee characteristics associated with acceptance status were selected based on descriptive statistics, and these need to be further examined for external validity.

## Supplementary material

For supplementary material accompanying this paper visit https://doi.org/10.1017/ash.2021.166. click here to view supplementary material
