# Supplementary material for: Severe acute respiratory coronavirus virus 2 (SARS-CoV-2) vaccine acceptance in employees in an integrated health system in the Midwest
Source: Antimicrob Steward Healthc Epidemiol. 2021 Jul 23;1(1):e14. doi: 10.1017/ash.2021.166 (PMC9495635; doi:10.1017/ash.2021.166)
Supplement: Supplementary file 1 [file ashsup.zip › S2732494X21001662sup002.pdf]

Follow-up Questionnaire for COVID-19 Survey of UPH Team Members

It has been a few weeks since you submitted information through the COVID-19 Survey of UnityPoint Health Team Members electronic questionnaire, and we are asking that you complete a brief update. You can still participate in the study, even if you plan to receive or have received the COVID-19 vaccine prior to your blood draw appointment. The antibody measured in this study is to a viral protein that is not included in the vaccine.

* Required

* This form will record your name, please fill your name.

1. COVID-19 Exposure:
   In the time period since you completed the electronic study questionnaire, have you had a significant exposure (within 6 feet for a cumulative period of >15 minutes in a day) to anyone with COVID-19 outside of work? *

   - ◯ Yes
   - ◯ No

2. Have you had a significant exposure (within 6 feet for cumulative period of > 15 minutes in a day) to a co-worker with COVID-19? *

   - ◯ Yes
   - ◯ No

3. Have you had a significant exposure (within 6 feet for any time period when all recommended PPE for the type of care provided was not utilized) to a patient with COVID-19? *

- ◯ Yes
- ◯ No

4. COVID-19 Diagnosis:
In the time period since you completed the electronic study questionnaire, have you been diagnosed with COVID-19? *

- ◯ Yes
- ◯ No

5. If yes, approximate date: *

Format: M/d/yyyy

6. How do you think you got COVID-19? *

- ◯ Home Exposure
- ◯ Community Exposure
- ◯ Work Exposure from Patient
- ◯ Work Exposure from Coworker
- ◯ Unsure
- ◯ Prefer Not to Answer

7. COVID-19 Vaccination:
In the time period since you completed the electronic study questionnaire, have you received the COVID-19 vaccine? *

- ◯ Yes
- ◯ No

8. If yes, approximate date: *

[ ]

Format: M/d/yyyy

This content is neither created nor endorsed by Microsoft. The data you submit will be sent to the form owner.

Microsoft Forms
