# Supplementary material for: Severe acute respiratory coronavirus virus 2 (SARS-CoV-2) vaccine acceptance in employees in an integrated health system in the Midwest
Source: Antimicrob Steward Healthc Epidemiol. 2021 Jul 23;1(1):e14. doi: 10.1017/ash.2021.166 (PMC9495635; doi:10.1017/ash.2021.166)
Supplement: Supplementary file 1 [file ashsup.zip › S2732494X21001662sup001.pdf]

COVID-19 Survey of UnityPoint Health Team Members

Participation in this study is voluntary. Your answers to this questionnaire will be kept anonymous. By completing this questionnaire, you are providing consent for your responses to be used in aggregate/summary with other collected responses. If you have questions or concerns about this study or questionnaire, please email UPH_COVID-19_Study@unitypoint.org (mailto:UPH_COVID-19_Study@unitypoint.org). Thank you for your participation!

* Required

* This form will record your name, please fill your name.

[ ]

Demographics

1. What is your age in years? *

- ◯ 18 - 20 years old
- ◯ 21 - 30 years old
- ◯ 31 - 40 years old
- ◯ 41 - 50 years old
- ◯ 51 - 60 years old
- ◯ 61 years old or older

2. What is your gender? *

- ◯ Man (includes transgender men)
- ◯ Woman (includes transgender women)
- ◯ Self-identify as Non-binary, Genderfluid, Gender Non-conforming
- ◯ Other
- ◯ Prefer Not to Answer

Work Characteristics

3. Do you spend most of your work time hours in the Des Moines region? *

- ◯ Yes
- ◯ No - If no, please stop now. Only team members working in the Des Moines region are eligible for this study at this time. Thank you!

4. Which best describes your work status? *

- ◯ Full Time
- ◯ Part Time

5. Which of the following best matches your occupation? *

- ◯ Administrative or Clerical
- ◯ Behavioral Health or Social Work
- ◯ Billing or Coding
- ◯ Dietary Services
- ◯ Engineering or Facilities
- ◯ Environmental Services
- ◯ Laboratory Personnel
- ◯ Nurse
- ◯ Nursing Assistant
- ◯ Occupational, Physical, or Speech Therapist
- ◯ Patient Services Representative
- ◯ Pharmacist
- ◯ Pharmacy Technician
- ◯ Phlebotomist
- ◯ Physician
- ◯ Physician Assistant or Nurse Practitioner
- ◯ Security
- ◯ Volunteer
- ◯ ______________ Other

6. Which of the following best describes your primary work setting? *

- ◯ Ancillary (RT, PT, OT, Pharmacy, Dietary, etc.)
- ◯ Emergency Department
- ◯ Home Care
- ◯ ICU
- ◯ Labor & Delivery
- ◯ Medical/Surgical Unit
- ◯ Non-Clinical or Administrative
- ◯ Outpatient Primary Care
- ◯ Outpatient Specialty Care
- ◯ Procedural (Cath Lab, Radiology, etc.)
- ◯ Surgery/OR/Endoscopy
- ◯ Other

7. Have you provided direct care to patients between March 1, 2020 and now? *

- ◯ No
- ◯ Yes

8. Which patient population do you typically work with? *

- ◯ Adult
- ◯ Pediatric
- ◯ Both

9. On average, how often do you provide direct patient care to patients known or suspected to have COVID-19? *

- ◯ Daily
- ◯ Multiple times per week
- ◯ Once per week
- ◯ Multiple times per month
- ◯ Once per month
- ◯ Less than once per month
- ◯ Never

Personal Protective Equipment (PPE) Use

We want to understand personal protective equipment (PPE) use in four direct patient care settings (COVID-19 patients vs. non-COVID-19 patients, during aerosol generating procedures vs. during non-aerosol generating procedures).

10. Do you provide cares while patients are undergoing any aerosol generating procedures (AGP)?
    This includes:
    - Intubation/Extubation
    - Nebulizer Treatment
    - Bag Valve Mask Ventilation
    - Cardiopulmonary Resuscitation
    - Suctioning of Open Airways
    - Tracheostomy Tube Insertion
    - Autopsy of Lung Tissue
    - Ventilator Disconnection
    - Non-Invasive Ventilation (BiPAP, CPAP)
    - High Frequency Oscillating Ventilation
    - Sputum Induction
    - Bronchoscopy
    - Surgery on Respiratory Tract *

    ○ No

    ○ Yes

11. In patients known or suspected to have COVID-19, what proportion of the time did you utilize the following types of PPE during aerosol generating procedures (AGP)? *

	Never	Sometimes	Frequently	Always	Not Applicable
Isolation/Procedure/ Surgical Mask	○	○	○	○	○
Eye Protection (includes face shield)	○	○	○	○	○
N-95	○	○	○	○	○
PAPR/CAPR	○	○	○	○	○

12. For COVID-19 negative or not suspected patients undergoing aerosol generating procedures (AGP), what proportion of the time did you utilize the following types of PPE? *

	Never	Sometimes	Frequently	Always	Not Applicable
Isolation/Procedure/ Surgical Mask	○	○	○	○	○
Eye Protection (includes face shield)	○	○	○	○	○
N-95	○	○	○	○	○
PAPR/CAPR	○	○	○	○	○

13. In patients known or suspected to have COVID-19 during cares that were NOT aerosol generating procedures (AGP), what proportion of the time did you utilize the following types of PPE? *

	Never	Sometimes	Frequently	Always	Not Applicable
Isolation/Procedure/ Surgical Mask	○	○	○	○	○
Eye Protection (includes face shield)	○	○	○	○	○
N-95	○	○	○	○	○
PAPR/CAPR	○	○	○	○	○

14. For COVID-19 negative or not suspected patients with cares that are NOT aerosol generating procedures (AGP), what proportion of the time did you utilize the following types of PPE? *

	Never	Sometimes	Frequently	Always	Not Applicable
Isolation/Procedure/ Surgical Mask	○	○	○	○	○
Eye Protection (includes face shield)	○	○	○	○	○
N-95	○	○	○	○	○
PAPR/CAPR	○	○	○	○	○

15. Did you provide direct patient care to patients known or suspected to have COVID-19 who required high flow nasal oxygen (such as AirVo, OptiFlo, VapoTherm, or Comfort Flow)? *

○ No

○ Yes

16. When caring for patients known or suspected to have COVID-19 who required high flow nasal oxygen, what proportion of the time did you utilize the following types of PPE? *

	Never	Sometimes	Frequently	Always	Not Applicable
Isolation/Procedures/ Surgical Mask	○	○	○	○	○
Eye Protection (includes face shield)	○	○	○	○	○
N-95	○	○	○	○	○
PAPR/CAPR	○	○	○	○	○

COVID-19 Exposure & Symptomatology

17. When unable to social distance, how frequently were you able to use a face covering? *

	Never	Sometimes	Frequently	Always
In the Workplace	○	○	○	○
In the Community	○	○	○	○

18. Have you had a known significant exposure (within 6 feet for a cumulate period of >15 minutes in a day) to anyone positive with COVID-19 outside of work? *

○ No

○ Yes

19. How likely do you think it is that you have had COVID-19? *

○ Extremely Unlikely

○ Unlikely

○ Equally Likely and Unlikely

○ Likely

○ Extremely Likely

20. Have you been tested for COVID-19 (utilizing nasal, nasopharyngeal, or oral sample)? *

○ No

○ Yes

21. Was the test result positive? *

◯ No

◯ Result Pending

◯ Yes

22. If your test result was positive, please enter the approximate date of the positive result. *

Format: M/d/yyyy

23. How do you think you got COVID-19? *

◯ Home Exposure

◯ Community Exposure

◯ Work Exposure from Patient

◯ Work Exposure from Coworker

◯ Unsure

◯ Prefer Not to Answer

24. If you feel comfortable, please describe in more detail the setting in which you think you got COVID-19.

25. Since March 1, 2020, have you had any of the following symptoms? Select all that apply. *

- [ ] Fever
- [ ] Shortness of Breath
- [ ] Cough
- [ ] Nasal Congestion/Rhinorrhea
- [ ] Sore Throat
- [ ] Headache
- [ ] Body Aches
- [ ] Fatigue
- [ ] Change in Smell or Taste
- [ ] None
- [ ] Other

26. Have you participated in a clinical trial to evaluate a COVID-19 vaccine? *

- ◯ Yes
- ◯ No

27. Have you received a COVID-19 vaccine outside of a clinical trial (for example, vaccine recently granted emergency use authorization by the FDA)? *

- ◯ Yes, one dose
- ◯ Yes, two doses
- ◯ No, but I intend to
- ◯ No, and I'm unsure if I will
- ◯ No, and I don't intend to

The following questions are required by the federal government for all COVID-19 tests.

This information will be provided to the lab team in order to meet their COVID-19 test reporting requirements.

28. First Name *

29. Last Name *

30. Date of Birth *

Format: M/d/yyyy

31. Gender *

- Male
- Female

32. Home Street Address (Include Apartment or Unit Number) *

33. City/Town of Residence *

34. State of Residence *

35. Zip Code of Residence *

36. Is this the first test (of any kind) you have had for COVID-19? *

- ◯ Yes
- ◯ No

37. Are you currently experiencing symptoms of COVID-19? If yes, please do no schedule your blood draw appointment until you are free of symptoms. *

- ◯ Yes
- ◯ No

38. Do you live in a congregate care setting (such as an assisted living facility, group home, or nursing home)? *

- ◯ Yes
- ◯ No

39. Are you currently pregnant? *

- ◯ Y (PPREG)
- ◯ N (NPREG)

ATTENTION!

Your next step will be to schedule an appointment for your blood draw. A link to use for scheduling will be sent to your UnityPoint Health email address.

This content is neither created nor endorsed by Microsoft. The data you submit will be sent to the form owner.

Microsoft Forms
